# Second-degree atrioventricular block induced by electrical stimulation of transcranial motor-evoked potential: a case report

**DOI:** 10.1186/s40981-024-00722-3

**Published:** 2024-06-12

**Authors:** Toru Murakami, Satoshi Tanaka, Ryusuke Tanaka, Mariko Ito, Takashi Ishida, Mikito Kawamata

**Affiliations:** Department of Anesthesiology and Resuscitology, Shinshu University School of Medicine, 3-1-1 Asahi, Matsumoto, Nagano 390-8621 Japan

**Keywords:** Motor-evoked potential, Arrhythmia, Paroxysmal atrioventricular block, Supine surgery, General anesthesia

## Abstract

**Background:**

Although several complications of transcranial motor-evoked potentials (Tc-MEPs) have been reported, reports of arrhythmias during Tc-MEP are very rare.

**Case presentation:**

A 71-year-old woman underwent transforaminal lumbar interbody fusion under general anesthesia, with intraoperative Tc-MEP monitoring. Preoperative electrocardiography showed an incomplete right bundle branch block but no cardiovascular events in her life. After induction of anesthesia, Tc-MEP was recorded prior to the surgery. During the Tc-MEP monitoring, electrocardiography and arterial blood pressure showed a second-degree atrioventricular block, but it improved rapidly at the end of the stimulation, and the patient was hemodynamically stable. Tc-MEP was recorded seven times during surgery; the incidence of P waves without QRS complexes was significantly higher than before stimulation. The surgery was uneventful, and she was discharged eight days postoperatively without complications.

**Conclusions:**

Our case suggests that electrical stimulation for Tc-MEP can cause arrhythmia. Electrocardiography and blood pressure must be closely monitored during Tc-MEP monitoring.

## Background

Spinal cord injury is a serious complication of spinal surgery, and guidelines recommend the use of transcranial motor-evoked potential (Tc-MEP) for its prevention [1, 2]. Although Tc-MEP is a relatively safe procedure, several complications have been reported, notably those associated with muscle contraction [2, 3]. Reports of arrhythmias occurring during Tc-MEP are rare [3–5] and the association or causal relationship between Tc-MEP and arrhythmias remains unknown. Herein, we report a case of reproducible second-degree atrioventricular block (AVB) related to Tc-MEP stimulation during transforaminal lumbar interbody fusion under general anesthesia.

## Case presentation

**Fig. 1:**
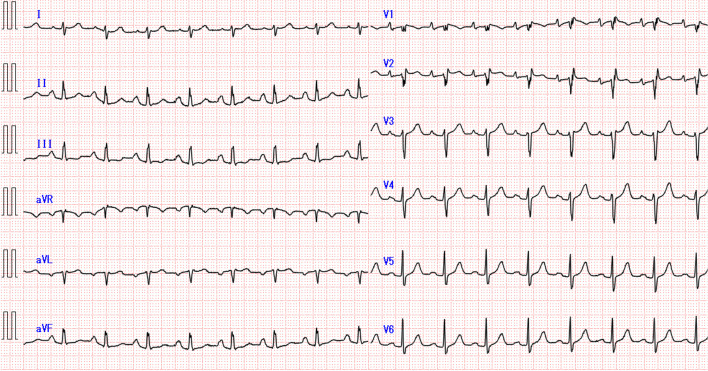
Preoperative electrocardiogram

A 71-year-old woman (height, 158 cm; weight, 47 kg; American Society of Anesthesiologists Physical Status II) with lumbar spinal stenosis was scheduled to undergo transforaminal lumbar interbody fusion under general anesthesia. Her medical history included rheumatoid arthritis and osteoporosis, for which she was treated with methotrexate and eldecalcitol. The patient had no history of syncope or other cardiac events. Blood tests showed mild anemia but no electrolyte abnormalities. Preoperative electrocardiography (ECG) showed sinus rhythm and an incomplete right bundle branch block (Fig. 1).

ECG, pulse oximetry, noninvasive blood pressure, invasive radial artery blood pressure (ABP), end-tidal carbon dioxide concentration, bladder temperature, and Bispectral Index (Medtronic-Covidien, Dublin, Ireland) were monitored in the operating room. General anesthesia was induced using propofol at a target concentration of 4 μg/ml and remifentanil at 0.18 μg/kg/min. After loss of consciousness, propofol was reduced to a target-controlled infusion of 3 μg/ml, 40 mg of rocuronium was administered, and the trachea was intubated. Anesthesia was maintained with target-controlled infusion of 2–3 μg/ml propofol to maintain a Bispectral Index value of 40–60, 0.1–0.3 μg/kg/min remifentanil, and an oxygen-in-air gas mixture. After induction of anesthesia, the patient was placed in the prone position.

Subsequently, Tc-MEP measurements were performed using a NuVasive NV-M5 monitoring system (NuVasive, San Diego, CA, USA). The NV-M5 monitoring system can use single-pulse stimulation and sets of sequential-pulse stimulation, which is characterized by sequential increases from 200 to 900 mA in 50 mA increments until the amplitude at the measurement site exceeds 50 μV, and requires several tens of seconds for a single Tc-MEP measurement. Transcranial electrical stimulation by train-of-five pulses (pulse width: 50 μs, interval of each stimulus: 2 ms) was applied through corkscrew electrodes to the C3 and C4 positions (internationals 10–20 electroencephalography system). To detect the compound muscle action potentials, seal-type electrodes were placed bilaterally on the deltoid, vastus medialis, tibialis anterior, biceps femoris, and gastrocnemius muscles.

**Fig. 2:**
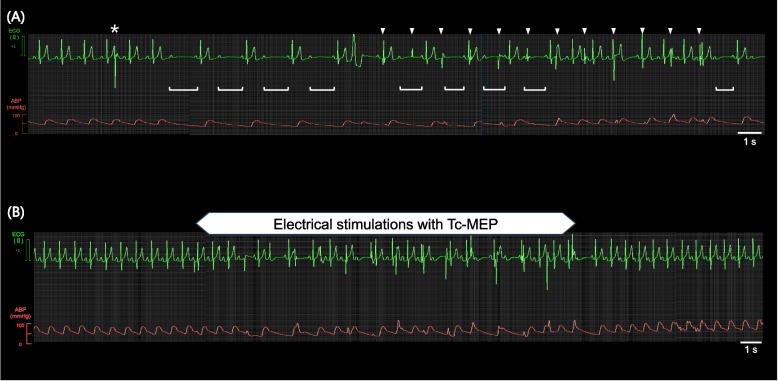
Examples of intraoperative second-degree atrioventricular block (AVB) associated with transcranial motor-evoked potential (Tc-MEP) stimulation. **A** Initial Tc-MEP stimulation and **B** a typical example of second-degree AVB associated with Tc-MEP stimulation. Lead II electrocardiogram and arterial blood pressure are shown. The asterisk indicates single Tc-MEP stimulation; white triangles indicate sequential Tc-MEP stimulation; white underlines represent QRS complex dropout after the P wave, and the underlined indicate consecutive P wave

A twitch test (four train-of-five pulses of 45 mA) was performed after muscle relaxation was antagonized with 190 mg sugammadex. Thereafter, an occasional second-degree AVB appeared but did not persist. Approximately 5 min after the twitch test, a single Tc-MEP stimulation (train-of-five pulses at 900 mA) and sequential Tc-MEP stimulation at baseline were performed. The frequency of second-degree AVB increased with stimulation (Fig. 2A). As the second-degree AVB improved briefly after Tc-MEP stimulation, and the patient's ABP decreased only slightly, we decided to perform surgery after preparing for transcutaneous pacing. Typical intraoperative ECG and ABP waveforms of second-degree AVB that appeared to be associated with Tc-MEP are shown in Fig. 2B. Throughout the operation, Tc-MEP measurements were performed seven times. However, the first two stimulations were performed at intervals of approximately 10 s for the baseline setting, and we were unable to determine the pre- and post-stimulation effects. Thus, excluding these two stimulations, the total stimulation duration of the five stimulations was approximately 112 s, with a minimum current of 200 mA and maximum current of 650 mA. For these five stimulations, the P waves and QRS complex for 20 s before stimulation, during stimulation, and 20 s after stimulation are shown in Table 1. The average incidence of second-degree AVB with Tc-MEP stimulation was 0% 20 s before stimulation, 22.8% during stimulation, and 2.7% 20 s after stimulation. There was no decrease in the P waves per unit time with Tc-MEP stimulation.

**Table 1:** Number of P wave and QRS complex before, during, and after electrical stimulation of Tc-MEP

	Before stimulation of MEP			During stimulation of MEP			After the end of stimulation of MEP		
Stimulation for Tc-MEP	P wave	QRS complex	Missing QRS complex	P wave	QRS complex	Missing QRS complex	P wave	QRS complex	Missing QRS complex
3rd	28	28	0	24	18	6	30	29	1
4th	29	29	0	29	23	6	30	30	0
5th	28	28	0	33	22	11	29	29	0
6th	29	29	0	37	26	41	30	28	2
7th	28	28	0	35	33	2	29	28	1
Total	142	142	0	158	122	36	148	144	4

*Tc-MEP* transcranial motor-evoked potential

During surgery, the patient’s hemodynamic status was relatively stable, with a heart rate of 44–89 bpm, and an ABP of at least 80/57 mmHg. Statistical analysis was performed using GraphPad Prism (version 9.0; GraphPad Software, San Diego, CA, USA), and the frequency of missing QRS complexes during Tc-MEP stimulation was significantly increased compared with that before stimulation (*p* = 0.0089, Friedman test followed by Dunn’s multiple comparison test). The total duration of surgery and anesthesia was 250 and 376 min, respectively. Intraoperative blood loss was 100 ml, infusion volume was 2060 ml, urine volume was 910 ml, and no blood transfusion was performed.

Anesthesia and surgery were uneventful except for a second-degree AVB, and transcutaneous pacing was not performed. There was no significant decrease in the amplitude of intraoperative Tc-MEP measurements. Emergence from anesthesia was smooth, and the patient was extubated without difficulty in the operating room. The 12-lead ECG performed after extubation did not differ significantly from the preoperative ECG findings. The patient was monitored by a cardiologist in the general ward, and the patient’s condition remained stable with no recurrence of the second-degree AVB. No additional treatment for arrhythmia was deemed necessary, and she was discharged without complications on postoperative day eight.

## Discussion

A recent literature review reported that complications related to Tc-MEP occurred in 167 of 38,195 cervical spine surgeries, with an incidence of 0.43%. Most complications are associated with muscle contractions, and only two are associated with arrhythmia or bradycardia [3]. Other arrhythmic complications associated with Tc-MEP include bradycardia [4], cardiac arrest [3], and third-degree AVB [5]. However, there have been very few reports of arrhythmic complications with Tc-MEP, and the association or causal relationship between Tc-MEP and arrhythmias is currently unknown. This report describes a case of second-degree AVB induced by electrical stimulation with Tc-MEP. Second-degree AVB rapidly improved at the end of electrical stimulation, which was reproduced by multiple stimulations. This suggests that the electrical stimulation of Tc-MEP likely caused arrhythmia.

At present, it is not clear why Tc-MEP repeatedly induced second-degree AVB in the patient. Vagally mediated AVB is characterized by decreased sinus rate [6] and can be induced by surgical manipulation, vagus nerve stimulation devices for epilepsy [7], etc. In our case, no prolonged P-P interval was observed. Therefore, it is unlikely that bradycardia due to vagal hyperactivity caused induction of second-degree AVB. It is possible that the arrhythmia that occurred during Tc-MEP in our case may have been paroxysmal AVB. Paroxysmal AVB is characterized by a sudden change from 1:1 atrioventricular conduction leading to complete AVB. Paroxysmal AVB is classified as intrinsic, extrinsic vagal, and extrinsic idiopathic paroxysmal AVB, and the P-P cycle may be decreased, increased, or unchanged [8]. Intrinsic AVB has been suggested to be caused by the presence of bundle branch block and exercise [9]. The patient with a right bundle branch block on the preoperative ECG may have been prone to paroxysmal AVB from Tc-MEP. Further studies are required to elucidate the mechanism underlying Tc-MEP stimulation-induced arrhythmia.

In conclusion, we reported a case of reproducible second-degree AVB related to electrical stimulation with Tc-MEP. In this study, arrhythmias were reproducibly induced by Tc-MEP stimulation, suggesting that electrical stimulation of Tc-MEP is a likely trigger for arrhythmias. Although infrequent, electrical stimulation by Tc-MEP may cause arrhythmias in some patients, which should be recognized and monitored when a Tc-MEP examination is performed.

## Data Availability

Not applicable.

## Abbreviations

Tc-MEP: Transcranial motor-evoked potential
AVB: Atrioventricular block
ECG: Electrocardiogram
ABP: Artery blood pressure
